# Associations between social capital and maternal depression: results from a follow-up study in China

**DOI:** 10.1186/s12884-018-1673-9

**Published:** 2018-02-02

**Authors:** Chi Zhou, Weijun Zheng, Qi Yuan, Baodan Zhang, Hao Chen, Weijue Wang, Liu Huang, Liangwen Xu, Lei Yang

**Affiliations:** 1 Medical School, Hangzhou Normal University, 16 Xuelin Rd., Xiasha District, Hangzhou, 310036 China; 2 School of Basic Medical Science, Zhejiang Chinese Medical University, Hangzhou, 310053 China; 3 Research Division, Institute of Mental Health, Singapore, 539747 Singapore

**Keywords:** Antenatal and postpartum depression, Cognitive SC, Structural SC, The Edinburgh postnatal depression scale, Follow-up study

## Abstract

**Background:**

This study aims to investigate the association between social capital (SC) and depressive symptoms among Chinese primiparas at different time-points from their late pregnancy to postpartum.

**Methods:**

A total of 450 primiparas were recruited for the current study. The assessments were conducted at three different time-points: T1 – while the participants were recruited at their 30–36 weeks of pregnancy in the antenatal clinic in the maternity hospital in Zhejiang, China; T2 – at their 2nd or 3rd days in the wards after delivery; T3 – at week 6 to 8 after the delivery in the postpartum examination clinic. SC was measured by the 29-item SC scale; while depressive symptoms were measured by the Edinburgh Postnatal Depression Scale. The relationships between SC and depressive symptoms were explored separately at each of the three time-points.

**Results:**

The prevalence of depression among the primiparas was 25% at T1, 13.5% at T2 and 20.8% at T3, respectively. However, the score of SC and its components at three time-points followed an opposite ‘V’ direction, with the highest score at T2, following by T3 and T1. At T1, the analysis suggested that depressive symptoms among the primiparas were negatively correlated with their social trust and social network levels. At T2, only social trust was negatively associated with depression. While at T3, it is social trust and social participations that were significantly negatively associated with depression.

**Conclusions:**

SC was associated with depression at all three time-points during and after pregnancy. More attention should be given to SC in the maternal health promotion programs of community pregnancy health care management.

**Electronic supplementary material:**

The online version of this article (10.1186/s12884-018-1673-9) contains supplementary material, which is available to authorized users.

## Background

Maternal depression is a serious public health issue [1]. Depressive symptoms are marked by an extended sense of sad mood, loss of interest, tearfulness, sleep problem, restlessness, irritability, appetite disturbance, and even suicidal ideation or attempt [2]. Previous studies suggested that depressive symptoms were found among about 10% to 20% of mothers, and such symptoms can last for several months or even a year [3]. Depression can happen at any time throughout the whole pregnancy process, from early pregnancy to even after delivery [4]. Antenatal depression itself is an important risk factor for maternal depression at other time-points for pregnant women as well [4]. Furthermore, due to the unique role of mothers in caring their babies, previous studies suggested that children of depressive mothers are three times more likely to develop serious emotional problems [5, 6].

A lot of studies have been done to explore the risk factors of maternal depression [7–9]. Recent reviews suggested that SC is a protective factor for individuals’ mental health status and it could also significantly reduce the risk of antenatal and postpartum depression [10–12]. SC is defined as “features of social organization, such as trust, norms and networks that can improve the efficacy of society by facilitating coordinated actions” [13]. It comprises two components, namely the cognitive SC and the structural SC [14]. The cognitive component refers to the internally subjective aspects of SC that reflect people’s perceptions on the level of interpersonal trust, sharing, reciprocity and other norms [15, 16]. The structural capital is more about the externally objective dimension and is featured by behavioral expression of social network and individual participation or community activities [17]. Till date, most studies on SC are cross-sectional in nature. In order to further explore the relationship between SC and maternal depression, a follow-up study design was used in the current study with both cognitive and structural SC being measured at the individual level.

The fourth baby boom period in China is approaching, as a result the number of women at their reproductive age is increasing rapidly, with an increment of about 2 million per year [18]; and a majority of them are the first generation of babies after the one-child policy was implemented [18, 19]. This group is very special, in the sense that they are the only child in their family and as a result most of them are ‘spoiled’. Moreover, a majority of them will be parents for the first time. In the traditional Chinese culture, parturients should practice “sitting the month” after their delivery, a period where they should stay at home for about one or two months to recover after they discharge from the hospital [20, 21]. This practice could bring huge changes on the life style and social environment of the parturients during this period. On one hand, primiparas need to adjust to get used to their new roles as mothers; meanwhile, the focus of the whole family will suddenly move towards the baby. These changes could result in a sudden drop of emotional support perceived by the primiparas [22]. On the other hand, “sitting the month” will limit their social activities, which will ultimately lower their perceived social support levels [19, 22]. Other than the above-mentioned issues, a lot of families nowadays also need older generations’ input (e.g. grandparents of the baby) while taking care of the babies. This arrangement has a high potential to cause cross-generation conflicts, which poses another potential risk towards the primiparas. Thus it is not surprising that about 50% to 75% of primiparas in China had reported experiences of unstable mood, accompanying by different level of physical symptoms in China [19].

To the best of our knowledge, there are very few studies had explored the relationships between SC and maternal depression at different time-points throughout the pregnancy progress. Using a sample of Chinese primiparas, the current study aims to answer the above question through a follow-up study design. We hypothesized that SC level of primiparas will be negatively correlated with depression among Chinese primiparas [10–12].

## Methods

### Study design

This study was conducted from March to December 2016 in the largest maternity hospital in Zhejiang Province, China. New mothers admitted to the hospital during the recruitment period were approached by the study team. Those who were willing to join the study were then assessed for their eligibility. To be included in the study, the primiparas should meet the following criteria: 1) in the hospital for the prenatal checkup, 2) 18 years old and above, 3) being pregnant for 30–36 weeks pregnant, 4) planning to give birth and attend postpartum follow up at the hospital, 5) willing to participate in the study, 6) literate in Chinese, and 7) not suffering from cognitive deficit. A total of 450 subjects were recruited for the current study and following informed voluntary written consent. The participation was voluntary, and they were informed that ‘refusal to participate will not affect the care they are going to receive from the hospital’. This study was approved by the Ethics Board of the Hangzhou Normal on 4th March 2016 (reference no.: ISRCTN2016014).

Data were collected at three different time-points: 1) T1 - while the participants were recruited at their 30–36 weeks of pregnancy in the antenatal clinical; T2 - at their 2nd or 3rd days in the wards after the delivery; T3 - at week 6 to 8 after the delivery in the postpartum examination clinic. To enable easier administration, the survey was built using an e-platform, and a QR code was generated. By scanning this QR code using their mobile phones, individuals could access to the full questionnaire including the socio-demographics and scales to evaluate primiparas’ SC and depressive symptoms. And they could choose the time point (i.e. T1, T2 and T3) and completed the survey on their mobile phones. This QR code was stuck onto the casebook of each participant, enabling the data collectors to easily identify the participants while they were in the hospital. In the case that the primiparas forgot to fill in the survey, phone calls were made by the investigators to remind them to scan that QR code to fill in the e-questionnaire. More detailed information regarding the measurement scales were included as the following.

### Measurements

SC was measured by the Chinese version of SC Assessment Questionnaire (C-SCAQ) [23]. This questionnaire was developed by Zhou and colleagues among Chinese primiparas based on the World Bank’s SC Assessment Tool and Bian’s Chinese position Table [24, 25]. More details of the C-SCAQ and tis development and validation could be found in Zhou et al. [23]. The C-SCAQ can be used to measure primipraras’ SC on both the cognitive and structural domains. Cognitive SC includes the social trust (ST) and social reciprocity (SR) sub-scales. The ST sub-scale has 8 items in total, and it measures the generalized trust among colleagues, neighbors and strangers. The SR consists of 7 items, and assesses the reciprocity among colleagues, neighbors and strangers. The score for each item varies from 1 to 5. The structural SC domain covers social network (SN) and social participation (SP). SN is assessed using Bian’s Chinese position Table [25], which considers Chinese social characteristics by position generator method. SN includes network diversity (the sum of occupations of the social network members) score ranging from 0 to 20, upper reach ability (the highest prestige occupation scores of social network members) score ranging from 0 to 95, and network range (the highest prestige occupation scores minus the lowest score of social network members) score ranging from 0 to 94. The SP has 11 items, covering the activity types, motivation and involvement. The items of activity types and motivation were graded using a 5-point Likert scale, while involvement was coded with 0 (nonparticipation) and 1 (participation). Since the internal measurement units are not uniform, the standardized score of SN and SP were used. Higher score represents higher SC level. The Cronbach’ α of cognitive and structural SC dimensions of C-SCAQ were 0.773 and 0.902 respectively in the previous study [26].

Depressive symptoms were measured by the Chinese version of Edinburgh Postnatal Depression Scale (C-EPDS). The scale consists of 10 items with 4 alternative answers for each item. The score for each item varies from 0 to 3, with a maximum score of 30 [27]. C-EPDS has been tested and validated among Chinese pregnant women before [28]. Compared to the Diagnostic and Statistical Manual of Mental Disorder-IV diagnosis of major depression as reference, C-EPDS demonstrated good reliability with a cutoff point score of 9 and above (sensitivity 0.72, specificity 0.88) [27]. This scale has also been used in other Chinese studies and showed high reliability in measuring prenatal and postnatal women’s depression [29, 30]. In the current study following the evidences, a cut-off of 9 was used to indicate the status of having depressive symptoms.

### Statistical analyses

Data were entered through EpiData 3.1 and analyzed by SPSS 20.0 (SPSS Inc., Chicago, IL, USA). We firstly described the socio-demographic characteristics of the sample. The participants were divided into two groups based on EPDS scores: the Depression group (score ≥ 9) and the Non-depression group (score < 9). The SC was compared between these two groups using T tests. Separate binary logistic regressions were conducted to assess the relationships between SC and depression at each of the three time-points, with the C-EPDS score being the dependent variable, and SC factors being the independent variables. The covariates in every regression included: age, district, education level, character, planned pregnancy, incoming per month, delivery by cesarean section, relationship with husband, relationship with parents-in-law, and sleep. In all regressions, a two-side *p*-value below 0.05 was treated as statistically significant.

## Results

### Sample characteristics

A total of 450 primiparas were eligible to participate in the study at T1, and 41 were excluded from the study representing a response rate of 91.1%. Of the 41 primiparas who were excluded, 25 (60.9%) refused to participate, and 16 (39.0%) intended to give birth in other hospitals. At T2 and T3, 376 (91.9%) and 288 (70.4%) primiparas completed the follow-up assessment, respectively. In the end, 288 primiparas who completed all the three interviews were chosen as research subjects in this study.

The demographic characteristics of primiparas are presented in Table 1. More than half of the subjects aged between 18 and 29 years old (68.4%), and 61.1% were from urban areas. More than half of the subjects had undergraduate college degrees and above (69.8%), and 63.5% described themselves as introverted. Most of the subjects had a planned to pregnancy (85.8%), and 58.3% had a monthly income of more than 4500 CNY. One third of participants were delivered by cesarean section (30.9%). After delivery, most of the subjects reported good relationships with husband (92.0%), and parents-in-law (74.7%). 15.3% of them suffered from poor sleep after delivery.

**Table 1:** Sample characteristics

Variate	Category	Frequency	Percent
Age	18–29	197	68.4
	30–45	91	31.6
District	urban	176	61.1
	rural	112	38.9
Education level	secondary school and below	2	0.7
	senior high school	24	8.3
	junior college	61	21.2
	undergraduate college	160	55.6
	master degree and above	41	14.2
Character	extrovert	105	36.5
	introvert	183	63.5
Planned pregnancy	yes	247	85.8
	no	41	14.2
Incoming per month	less than 1500 RMB	11	3.8
	1500–3000 RMB	18	6.3
	3001–4500 RMB	91	31.6
	more than 4500 RMB	168	58.3
Delivery by cesarean section	yes	89	30.9
	no	199	69.1
Relationship with husband	good	265	92.0
	general	20	6.9
	poor	3	1.1
Relationship with parents-in-law	good	215	74.7
	general	66	22.9
	poor	7	2.4
Sleep	good	104	36.1
	general	140	48.6
	poor	44	15.3

### Prevalence, timing of depression and SC

**Fig. 1:**
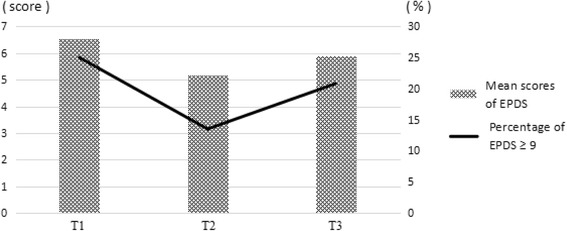
Timing of maternal depression. Mean scores of Edinburgh Postnatal Depression Scale and percentage of Edinburgh Postnatal Depression Scale ≥9 among Chinese primiparas at different time-points

Figure 1 shows 25% the prevalence of depressive symptoms of the participants. 25% of the primiparas were found to have antenatal depression at T1. 13.5% and 20.8% showed symptoms of postpartum depression at T2 and T3 after delivery, respectively. The mean scores of the EPDS were higher at T1 and T3, and lowest at T2.

**Fig. 2:**
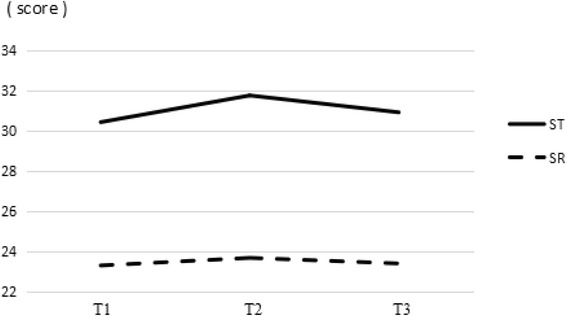
Timing of cognitive SC. The scores of social trust and social reciprocity at three different time-points. ST: social trust; SR: social reciprocity

**Fig. 3:**
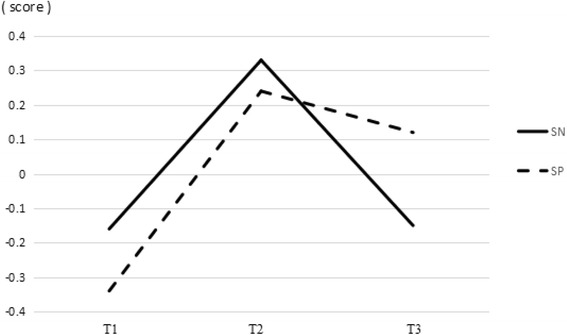
Timing of structural SC. The scores of social network and social participation at three different time-points. SN: social network; SP: social participation

The scores of cognitive and structural SC at three different time-points are presented in Figs. 2 and 3, and the curves had opposite directions comparing to that of the EPDS in Fig. 1. The mean scores of SC at different time-points are presented in Table 2. The cognitive and structural SC levels were lower at T1 and T3, and highest at T2.

**Table 2:** The mean scores of SC at three different time-points

Variable	T1		T2		T3	
	Mean	SD	Mean	SD	Mean	SD
Cognitive SC						
Social trust	30.44	4.21	31.77	4.61	30.93	5.02
Social reciprocity	23.20	4.36	23.68	4.65	23.40	5.03
Structural SC						
Social network	− 0.16	2.35	0.33	2.53	−0.15	2.51
Social participation	−0.34	2.22	0.24	2.54	0.12	2.88

### Association between SC and depression

The association between SC and maternal depression are presented in Table 3. The ST, SR and SP mean scores of Depression group were significantly lower than those of the Non-depression group at all three assessments (*p* < 0.05). The mean SN score of the Depression group was significantly lower than that of the Non-depression group at T1 (*p* < 0.01).

**Table 3:** SC comparison between depression and non-depression group

Variable	Depression group		Non-depression group		*t* test	
	Mean	SD	Mean	SD	*t*	*p*
Cognitive SC						
Social trust (T1)	28.72	4.17	31.01	4.07	4.11	< 0.01
Social trust (T2)	29.38	4.29	32.15	4.56	3.55	< 0.01
Social trust (T3)	28.62	3.87	31.54	5.12	4.13	< 0.01
Social reciprocity (T1)	21.79	3.80	23.67	4.44	3.22	< 0.01
Social reciprocity (T2)	22.00	3.64	23.95	4.74	2.97	< 0.01
Social reciprocity (T3)	20.85	4.22	24.07	5.02	4.56	< 0.01
Structural SC						
Social network (T1)	−0.86	2.39	0.08	2.30	2.97	< 0.01
Social network (T2)	− 0.42	2.60	0.44	2.51	1.98	0.05
Social network (T3)	−0.54	2.45	−0.04	2.52	1.39	0.17
Social participation (T1)	−0.82	2.13	−0.18	2.23	2.17	0.03
Social participation (T2)	−0.95	2.05	0.43	2.57	3.20	< 0.01
Social participation (T3)	−1.06	2.63	0.44	2.86	3.66	< 0.01

After controlling for the exogenous variables, adjusted associations between antenatal depression and SC are displayed in Table 4. The model at T1 shows that ST (*β* = − 0.90, *p* < 0.01) and SN (*β* = − 0.83, *p* < 0.01) were significantly negatively correlated with antenatal depression. The model at T2 shows that ST (*β* = − 0.76, *p* = 0.04) was significantly negatively correlated with postpartum depression. The model at T3 shows that ST (*β* = − 0.85, *p* = 0.04) and SP (*β* = − 0.74, *p* = 0.04) were significantly negatively correlated with postpartum depression.

**Table 4:** Adjusted associations between SC and depression

Variables	*β*	SE	*df*	*P*
T1 model				
Social trust	−0.90	0.34	1	< 0.01
Social reciprocity	−0.41	0.34	1	0.24
Social network	−0.83	0.29	1	< 0.01
Social participation	−0.38	0.33	1	0.24
T2 model				
Social trust	−0.76	0.41	1	0.04
Social reciprocity	−0.76	0.46	1	0.10
Social network	−0.17	0.36	1	0.65
Social participation	−0.72	0.40	1	0.05
T3 model				
Social trust	−0.85	0.41	1	0.04
Social reciprocity	−0.46	0.40	1	0.25
Social network	−0.26	0.31	1	0.42
Social participation	−0.74	0.36	1	0.04

## Discussion

### Depression status of primiparas

This study showed that the prevalence of depressive symptoms of Chinese primiparas throughout the pregnancy process following a “V” shape, and this is similar to findings from a previous study in Greece [27]. The highest prevalence of EPDS was at T1, and the lowest one at T2. EPDS scores at T1 were higher than T3. However, studies in China tended to pay more attention to the period of 6–8 weeks postpartum among the primiparas [31–33]. We observed that the prevalence of depression was highest at the late pregnancy among our primiparas sample. This suggested a potential high cost-effectiveness if timely interventions could be implemented at this time-point.

In the literature, one cohort study by Milgrom et al. in UK showed that depression scores were 6.72 at 32 weeks of pregnancy, and higher than 5.84 at 8 weeks postpartum [34]. Rich-Edwards et al. found that the prevalence of depressive symptoms was 9% at mid-pregnancy and 8% at postpartum [35]. These are similar to our study, but some other studies in the literature have different findings. O’Hara et al. found that the highest depression rate was 12% at 9 weeks postpartum, and 9% at the second trimester pregnancy [36]. A 6-month follow-up study indicated that 6.8% women were found to have postpartum depression at the end of first week after delivery, and the prevalence were 12.5%, 9.0%, and 4.9% at the end of the first, third, and sixth month respectively [27]. Josefsson et al. showed that the prevalence of depressive symptoms during late pregnancy was 17%; in the maternity ward 18%; 6–8 weeks postnatally 13%; and 6 months postnatally 13% in Sweden [37].

### SC status of primiparas

Results from the current study suggested that the SC scores throughout the pregnancy process followed an upside-down “V” shape. The curve is opposite to that of the EDPS scores, and consistent with our hypothesis. Firstly, the highest social capital level was at T2. Usually, primiparas will leave the hospital 3 to 5 days after delivery, thus they were still in the hospital at the 2nd to 3rd days after delivery. During this period, their husband or parents will carefully take care of them, and their relatives, friends and colleagues will visit them and give red envelopes or newborn gifts [38]. Those social interactions and gifts might give them with greater support and comfort. Moreover, the happiness levels for new mothers are normally very high at this time-point due to the joyfulness they would experience in the sense of having a baby.

Secondly, the results shows that SC level went down after they discharged from hospital, and moved to a relatively low level at T3. In China, primiparas should be confined at home for one full month of convalescence after discharge from the hospital [39]. This practice might change their normal social environment. Moreover, their psychological status might be largely affected by the attitude of family caregivers [38]. Similar finding was reported by another study in China, suggesting that pregnant women had the highest level of social support level at 1 week postpartum, and decreased afterwards [38]. The government needs to encourage regularly postpartum visits to improve the primary maternal health management, and doctors can provide them with more professional supports.

Thirdly, it was beyond our expectation that the lowest SC level was at T1. It may be the case that pregnant women at this time-point are too clumsy to move around [40, 41]. This will limit their social activities, and result in loss of face-to-face contacts with friends and acquaintances [40, 41].

Another important finding of our study is that, the two curves of structural SC had larger fluctuations than curves of cognitive SC. Through interviews with primiparas, we learnt that a lot of the primiparas took part in training such as parenting classes provided by the maternity hospital before delivery to better prepare for their coming first-time delivery. Through these activities, they got the opportunities to meet doctors, nurses, and other new mothers; and make friends with others, as a result to expand their social networks [38]. Their activity levels and networks would decline rapidly during the confinement. However, ST and SR which require longer time to develop would stay. According to the blood relationship culture in China, they specifically trust their family members [42]. During the whole pregnancy and childbirth period, their family members will always be around with them. Although their cognitive SC level might be influenced by the confinement, it is still more stable than the structural SC.

### Influence of SC on antenatal and postpartum depression among primiparas

This study examined the effect of SC on antenatal and postpartum depression among Chinese primiparas, and analyzed the relationship between SC and maternal depression. In previous review articles, few studies carried out follow up research on the association between SC factors and maternal depression among pregnant women [43, 44]. Our study includes four dimensions of SR, ST, SN, and SP from cognitive and structural SC at the individual level, and is also the first follow-up study in China with such a sample size.

The current study found that the Non-depression primiparas had higher SC levels (ST, SR, SN and SP) than Depression group at all three time-points. Similar findings were also reported in a longitudinal study by Lamarca et al. [43]. They investigated the associations between individual SC (social support and social networks) and consistent self-rated health in women between the first trimester of pregnancy and six months postpartum, and found that the good self-rated health group had higher scores of SC than the poorer group [43].

In our study, ST had strong association with depression at all the three time-points. This is consistent with previous studies. Fujiwara and Kawachi found that perceptions of higher levels of cognitive SC (social trust) were associated with lower risks of developing major depression among adults during the 2–3 year follow-up [44]. A prospective analysis also indicated that low interpersonal trust appears to be an independent risk factor for new-onset and long-term depression in South Korea [45]. A potential explanation could be that interpersonal trust can provide emotional support to help primiparas to deal with their stress [45, 46].

It is interesting that SN has a strong association with antenatal depression at T1, and SP was associated with postpartum depression at T3. It may be the case that primiparas will get to know new mothers and nurses and expand their networks through pregnant women school activities as mentioned above. These networks can provide emotional support for primiparas on delivery and parenting, and it might also be beneficial to reduce their depression level and needs to be further verified [34]. After delivery, appropriate participation is helpful to alleviate depression, but the traditional confinement seems to be an obstacle for SP. Armstrong and Edwards showed that mothers in the pram-walking intervention group improved their fitness levels and reduced their level of depressive symptomatology significantly more than the social support group [47]. Zhang also indicated that taking active parts in social activities if physical conditions permit can reduce postpartum anxiety [48].

There are a few limitations for the current study. Firstly, this study examines the relationship between SC and peripartum depression at only three time-points: 30–36 weeks of pregnancy, 2–3 days and 6–8 weeks postpartum. So we might miss the highest point of depression and the lowest point of SC level. If the survey can be extended to include the first trimester to 6 months after delivery, and the results of observation might be more intact. Secondly, EPDS is a self-report tool to measure mood, and there might overpathologising motherhood [49]. This phenomenon has a certain effect on the results. Thirdly, although a follow-up design was used, regression analyses to explore the relationships between SC and maternal depression were conducted separately at each time-point. Fourthly, given that the study participants were from Zhejiang province, this might limit the generalization of the study findings. Lastly, personality of primiparas was measured by a single question ‘Would you describe yourself as extroverted or introverted?’, without using a scale. More studies are still needed to confirm whether the findings from the current study will be applicable nation-wide.

## Conclusions

We examined the association of SC and maternal depression among Chinese primiparas. The prevalence of EPDS had a “V” type distribution and SC form an upside-down “V”. The two curves of structural SC had larger fluctuations than curves of cognitive SC. The SC level of Depression group was lower than Non-depression group at all three time-points. ST and SN were significantly associated with the depression of primiparas at T1, ST was associated at T2, and ST and SP were associated at T3. For future maternal health education and promotion programs, more attention could be given to SC improvement in the community pregnancy health care management.

## Additional file

Additional file 1: Survey data: Data of Social capital and depressive symptoms scores were collected among Chinese primiparas at different time-points from their late pregnancy to postpartum. (XLSX 195 kb)

## Abbreviations

C-EPDS: Chinese version of Edinburgh postnatal depression scale
C-SCAQ: Chinese version of social capital assessment questionnaire
PPD: Postpartum depression
QR Code: Quick response code
SC: Social capital
SN: Social network
SP: Social participation
SR: Social reciprocity
ST: Social trust
